# Supplementary material for: Mycobacterium tuberculosis Central Metabolism Is Key Regulator of Macrophage Pyroptosis and Host Immunity
Source: Pathogens. 2023 Aug 30;12(9):1109. doi: 10.3390/pathogens12091109 (PMC10535942; doi:10.3390/pathogens12091109)
Supplement: Supplementary file 1 [file pathogens-12-01109-s001.zip › pathogens-2555072-supplementary.pdf]

pathogens

MDPI

Supporting Information

Materials and Methods

Mycobacteria strains and culture conditions.

A complete list of mycobacteria strains used in this study is presented in Table S1. For routine growth, mycobacteria were grown in Middlebrook 7H9 broth (Difco, Sparks, MD) supplemented with 10% (v/v) OADC enrichment (BD Diagnostic Systems), 0.5% (v/v) glycerol, and 0.05% (v/v) tyloxapol or Middlebrook 7H10 agar (Difco, Sparks, MD) containing 10% (v/v) OADC enrichment, and 0.5% (v/v) glycerol. Selection antibiotics were used at the following concentrations: kanamycin 40 μg mL^{-1}, hygromycin 75 μg mL^{-1}, apramycin 40 μg mL^{-1}. Auxotrophic *M. tuberculosis* strains were grown in the presence of D-leucine 50 μg mL^{-1} and/or D-pantothenate 25 μg mL^{-1}, as needed. In all cases, mycobacteria were grown at 37 °C , shaken at 100–125 rpm.

Monocyte / MΦ and cell culture conditions.

THP-1 cells (ATCC# TIB-202), a human acute monocytic leukemia cell line, were grown in suspension using complete RPMI media [RPMI (Invitrogen) supplemented with 0.5% MEM essential amino acids (Invitrogen cat # 11130-051), 0.5% MEM nonessential amino acids (Invitrogen cat # 11140-050), 10 mM HEPES (Invitrogen), 55 μM β-mercaptoethanol, 10% heat-inactivated fetal calf serum (Gemini)]. Cells were maintained at a concentration of $2 \times 10^5 – 8 \times 10^5$ cells mL^{-1} in humidified 37 °C / 5% CO_2 incubators.

For differentiation into adherent MΦ, THP-1 cells were counted, centrifuged at 1500 rpm for 10 min and resuspended in complete RPMI media containing 50 nM phorbol 12-myristate 13-acetate (PMA, Sigma cat # P8139) at a concentration of 1×10^6 cells mL^{-1}. Cells were then seeded into Costar microtiter plates and incubated ≥ 16 hr prior to experiments.

For human primary cell experiments, CD14+ monocytes were isolated from peripheral blood mononuclear cells (PBMC) by using a positive selection CD14 microbeads and MACS column according to the manufacturer's protocols (Miltenyi Biotech cat # 130-050-201). Selected monocytes were resuspended in complete RPMI media containing 10% AB Human serum (Gemini cat # 100-512) seeded into a 48 well microtiter plate for 7 days prior to infection. Cells were maintained at a concentration in humidified 37 °C / 5% CO_2 incubators. Cell densities were then adjusted to 1×10^6 cells mL^{-1} prior to experiments.

Live-cell Hoechst 33258 staining of THP-1 macrophage

3 days post-infection, the media from mycobacteria-infected THP-1 monolayers were stained in 1μg mL^{-1} Hoechst 33258 (Sigma cat # B2883), 1X PBS added. Monolayers were incubated at 37 °C for 10 min, then visualized by fluorescence microscopy under 200× magnification in the blue channel (BP330-385 filter). Cells undergoing cell death would take up the Hoechst nuclear stain; condensed or fragmented nuclear morphology suggested apoptotic or pyroptotic (but not necrotic) cell death.

Transposon mutagenesis of mycobacteria and screen methodology

High-titer phage derived from phasmid phAE180, and containing *mariner* transposon construct pMycoMar [104,105], was used to mutagenize *M. bovis* BCG strain mc^26455 by transduction. For construction of the transposon mutant library plates, 10,000 transposon mutants (individual Km^R colonies) were picked and inoculated into separate wells of 96-well microtiter plates, containing of 7H9 broth with 40 μg mL^{-1} kanamycin. These transposon library master plates were incubated at 37 °C until saturated.

96-well mc^26455 transposon mutant library was subcultured to an OD_{600nm} of approximately 0.2 and used to infect THP-1 macrophage in 96-well plate format. THP-1 cells were prepped for infection as described above. Monolayers were infected with the mc^26455

transposon mutant library by multichannel pipette using the methods described above. After 4 days infection, THP-1 monolayers were live-cell stained with Hoechst stain and visualized by microscopy (see above). Wells showing increased Hoechst staining were marked as candidate *fdr* (*f*unctioning *d*eath *r*epressor) mutants for further study.

The *mariner* transposon used contains an oriR6Kγ that allows for the recovery of transposon-chromosome junctions as replicating plasmids. To map transposon interstation sights, transposon mutant genomic DNA was digested with BssHII, ligated, and transformed into DH5α-λpir cells, which support oriR6Kγ replication. Recovered plasmids were sequenced, and sequence data compared to the *M. tuberculosis* H37Rv annotated genome [106] by BLASTn (NCBI) to identify transposon insertion sites.

Terminal deoxynucleotidyl transferase dUTP nick end labeling (TUNEL).

In Situ Cell Death Detection Kit, Fluorescein (Roche) was used to detect DNA fragmentation in adhered cells by microscopy. Briefly, 4×10^5 THP-1 cells were seeded onto 12 mm glass coverslips in 24 well plates, following differentiation protocol above. Infections with various mycobacteria strains were done as described, and 4 days post-infection, coverslips were fixed in 2% paraformaldehyde for 30 min at 37ºC. TUNEL staining protocol was done, according to the manufacturer's instructions. Stained monolayers were visualized by fluorescence microscopy using 200× magnification under the green channel (S492/18× filter). DNA fragmentation would result in TUNEL positive cells. For each sample, cells were counted from three different fields; with 100 cells counted per field.

Detection of caspase-dependent cell death versus necrosis.

FAM-FLICA *in vitro* poly-caspases assay kit (Immunochemistry Technologies) was used to detect active caspases in infected THP-1 cells by microscopy, and distinguish caspase-dependent cell death from necrosis. Briefly, 4×10^5 THP-1 cells were seeded onto 12 mm glass coverslips in 24 well plates, following differentiation protocol above. Infections with various mycobacteria strains were done as described, and 3 days post-infection, coverslips were stained propidium iodide and FAM-VAD-fmk, according to the manufacturer's instructions for adhered cells. Stained monolayers were visualized by fluorescence microscopy using 200× magnification under the green and red channels (S492/18×and S572/23× filters, respectively). Necrotic cell death would result propidium iodide positive and caspase negative cells, while caspase-dependent cell death would result in caspase positive and either propidium iodide negative (apoptosis) or positive (pyroptosis) cells. For each sample, cells were counted from three different fields; with 100 cells counted per field.

Specialized transduction for the generation of precise null mutants.

Allelic exchange substrate for deletion of Rv3727 were generated by cloning PCR products containing left and right homologous arms to the gene of interested, into Van91I-digested p0004S (Hyg^R AES cloning vector, T. Hsu and W.R. Jacobs, unpublished). Primers used were: Rv3727_LL_AlwNI [5′ TTT TTT TTC AGA AAC TGC GGT TCG TCC TCG GCT ACA C 3′], Rv3727_LR_AlwNI [5′ TTT TTT TTC AGT TCC TGA TCG GCA CTG GAA GTT CTT TGA TGT CTC ACT GAG GTC TCT GGC AAT CAC GAC GTG GGT ATC 3′], Rv3727_RL_Van91I [5′ TTT TTT TCC ATA GAT TGG ACC AAT CGT CCA TAC TAG GAC GAG TGT CTG GTC TCG TAG CCA CCT GAC CGA CGT GGA 3′], Rv3727_RR_Van91I [5′ TTT TTT TTC CAT CTT TTG GAA CCA GTT CCC CGA CGC TTT 3′]. Phasmids for allelic exchange were generated by cloning PacI linearized AES into the unique PacI site of phAE159 [107].

High-titer phage derived from allelic exchange phasmids were used to transduce *M. bovis* BCG Danish and *M. tuberculosis* H37Rv *ΔpanCD ΔleuCD* strains . One milliliter of high-titer phage was added to 1 mL of cells, and transductions incubated at 37 °C for 24

hr. Following this, each transduction was plated on two 7H10 agar plates containing 75 μg mL^{-1} hygromycin. Plates were incubated 3 weeks at 37 °C.

Hyg^R transductants were inoculated into 5 mL 7H9 broth containing 75 μg mL^{-1} hygromycin and grown to saturation. Transductants were screened for gene deletion by PCR of genomic DNA, using primers: Rv3727_LL_noRE [5′ CGG TTC GTC CTC GGC TAC AC 3′], Rv3727_RR_noRE [5′ AAC CAG TTC CCC GAC GCT TT 3′].

Animal studies

6–8 week-old female wild type C57BL/6 mice were obtained from Jackson Laboratories. All mice were maintained in specific pathogen-free conditions. All procedures involving the use of animals were in compliance with protocols approved by the Albert Einstein College of Medicine Institutional Animal Care and Use Committee (IACUC Protocol # 20090309) and Biosafety Committees.

RNA purification from mycobacteria.

RNA was extracted and purified using the Fast RNA prep and QIAGEN RNeasy systems. Between 10^8 – 10^9 cfu of mycobacteria were resuspended in 1 mL Buffer RLT containing β-mercaptoethanol (RNeasy kit, QIAGEN), and aliquoted into a Lysing Matrix B tube (Qbiogene). Samples were processed in a FastPrep FP120 machine (Qbiogene) 1 time for 45 sec at speed 6, and immediately placed on ice. After 1 min incubation, supernatants were mixed with 100% ethanol to a final concentration of 45%, and processed according to the standard instructions and reagents provided with the QIAGEN RNeasy kit.

For RNA isolation from intracellular mycobacteria, to isolate approximately 10^9 intracellular bacteria from infected THP-1 cells, infection protocol described above was scaled up to T-175 flasks containing 3×10^7 MΦ per flask, with 9 flasks infected in triplicate per mycobacteria strain. THP-1 cells were infected with mycobacteria strains at an MOI of 5:1. 3 days post infection, intracellular bacteria were harvested monolayers using the protocol from [108].

RNA quantity and quality was assessed with a NanoDrop-1000 micro-volume UV-Vis spectrophotometer (Thermo Scientific). RNA samples were aliquoted into 5 μg portions and stored at −80 °C until needed.

RNA purification from mycobacteria-infected THP-1 cells.

RNA was extracted and purified using the QIAGEN RNeasy kit, from 2×10^6 THP-1 cells that had been previously infected by mycobacteria as described above. 3 days post-infection, media was aspirated from THP-1 cells and 1 mL RNAprotect Cell Reagent (QIAGEN) added. THP-1 cells were resuspended in Buffer RLT containing β-mercaptoethanol, homogenized using the QIAshredder spin column. Absolute ethanol was added to the lysate to a final concentration of 50%, and samples processed according to the standard instructions and reagents provided with the QIAGEN RNeasy kit. RNA quantity and quality was assessed with a NanoDrop-1000 micro-volume UV-Vis spectrophotometer (Thermo Scientific). Quality was also checked by Agilent 2100 Bioanalyzer total RNA assay, using a Nano RNA Chip. RNA samples were aliquoted into 10 μg portions and stored at −80 °C until needed.

Table S1. Bacterial strains used in this study.

Strain	Genotype	Reference
mc²6206	*M. tuberculosis* H37Rv Δ*panCD* Δ*leuCD*	gift of M.H. Larsen and K.E. Biermann
mc²6887	*M. kansasii* Hauduroy ATCC 12478	[109]
mc²6450	*M. bovis* BCG Danish strain 1331	[110]
mc²6455	mc²6450 Δ*ureC::fbpBp/fbpB*$_{ss}$'-'*pfoA*(G137Q)$_{\Delta ss}$	[31]
mc²6405 [a]	mc²6455 Rv0947c::*magellan4 (fdr5)*	†
mc²6406	mc²6455 *arcA*::*magellan4 (fdr16)*	†
mc²6408 [a]	mc²6455 Rv1831::*magellan4* (*fdr2*)	†
mc²6409 [a]	mc²6455 Rv1953::*magellan4;* TB16.3::*magellan4 (fdr12)*	†
mc²6410	mc²6455 *tmk-mtrA*::*magellan4 (fdr17)*	†
mc²6412 [a,b]	mc²6455 Rv3727::*magellan4* *(fdr8)*	†
mc²6413 [a,b]	mc²6455 Rv3727::*magellan4* *(fdr15)*	†
mc²6414	mc²6455 *tcrX*::*magellan4 (fdr4)*	†
mc²6417 [a]	mc²6455 Rv1204c::*magellan4 (fdr11)*	†
mc²6427	mc²6450 ΔRv3727::γδ*res-sacB-hyg*-γδ*res*	†
mc²6444	mc²6455 ΔRv3727::γδ*res-sacB-hyg*-γδ*res*	†
mc²6891 [c]	mc²6405 *glyV*::1A7 (integrating cosmid containing *M. tuberculosis* genome region ′Rv0946c-Rv0977c′, Hyg^R)	[111] †
mc²6892 [c]	mc²6406 *glyV*::2H9 (integrating cosmid containing *M. tuberculosis* genome region ′Rv0982-Rv1011′, Hyg^R)	[111] †

mc²6893 [c]	mc²6408 *glyV*::4G4 (integrating cosmid containing *M. tuberculosis* genome region Rv1813c′-′Rv1837c, Hyg^R)	[111] †
mc²6894 [c]	mc²6409 *glyV*::7C12 (integrating cosmid containing *M. tuberculosis* genome region Rv2180c′-′Rv2211c, Hyg^R)	[111] †
mc²6895 [c]	mc²6410 *glyV*::3C12 (integrating cosmid containing *M. tuberculosis* genome region Rv3237c′-Rv3262′, Hyg^R)	[111] †
mc²6896 [c]	mc²6410 *glyV*::6G2 integrating cosmid containing *M. tuberculosis* genome region Rv3225c-′Rv3257c, Hyg^R)	[111] †
mc²6897 [c]	mc²6411 *glyV*::1G9 (integrating cosmid containing *M. tuberculosis* genome region Rv1326′-′Rv1356c, Hyg^R)	[111] †
mc²6898 [c]	mc²6411 *glyV*::8C3 (integrating cosmid containing *M. tuberculosis* genome region ′Rv1315-′Rv1343c, Hyg^R)	[111] †
mc²6899 [c]	mc²6413 *glyV*::5C4 (integrating cosmid containing *M. tuberculosis* genome region ′Rv2825c-Rv2787, Hyg^R)	[111] †
mc²6900 [c]	mc²6413 *glyV*::1G3 (integrating cosmid containing *M. tuberculosis* genome region Rv3707c′-Rv3731′, Hyg^R)	[111] †
mc²6901 [c]	mc²6414 *glyV*::6H12 (integrating cosmid containing *M. tuberculosis* genome ′Rv3736-′Rv3767c, Hyg^R)	[111] †
mc²6902 [c]	mc²6417 *glyV*::6B9 (integrating cosmid containing *M. tuberculosis* genome region ′Rv1187-Rv1223′, Hyg^R)	[111] †
mc²6903 [c]	mc²6417 *glyV*::8D9 (integrating cosmid containing *M. tuberculosis* genome region ′Rv1182-Rv1208′,	[111] †

	Hyg^{R})	
mc^{2}6904	mc^{2}6206 ΔRv3727::γδ*res-sacB-hyg*-γδ*res*	†

[a] Rv ORF number refers to the *M. tuberculosis* ortholog of the *M. bovis* BCG gene identified by transposon mutagenesis.

[b] Two independent transposon hits identified for the same ORF.

[c] Indicates a cosmid integrant (*M. tuberculosis* genomic cosmid library); confirmed by PCR from phenol/chloroform-extracted culture boil-preps.

† Constructed as part of this study.

Table S2. Genes significantly over-represented 2-fold or more in intracellular *fdr8* compared to intracellular mc^{2}6455 (wild type), 3 days post THP-1 cell infection.

ORF	Gene Function ^{a}	Fold change compared to wild type
MT0023	hypothetical protein	2.59
MT0530	glutamyl-tRNA reductase	4.27
MT0955	phosphate abc transporter, phosphate-binding protein	4.24
MT1479	hypothetical protein	2.58
MT1838.1	hypothetical protein	2.65
MT1839.1	hypothetical protein	3.54
MT3137	ribonucleotide-diphosphate reductase alpha subunit	3.24
MT3615.1	PE-PGRS family protein; gene contains a frame shift	3.03
MT3693	hypothetical protein	2.61
Rv0005	DNA topoisomerase IV subunit b	4.10
Rv0007	possible conserved membrane protein	2.62
Rv0009	iron-regulated peptidyl-prolyl cis-trans isomerase a PpiA	4.70
Rv0013	para-aminobenzoate synthase component II	3.01
Rv0014c	transmembrane serine/threonine-protein kinase b PknB	3.86
Rv0019c	hypothetical protein	3.06
Rv0020c	hypothetical protein	5.09
Rv0053	30s ribosomal protein s6	2.73
Rv0055	30s ribosomal protein s18	3.20

Rv0125	probable serine protease PepA (MTB32a)	4.80
Rv0171	mce-family protein Mce1C	5.95
Rv0172	mce-family protein Mce1D	4.76
Rv0173	possible mce-family lipoprotein LprK; Mce1E	6.33
Rv0175	probable conserved mce associated membrane protein	2.56
Rv0199	probable conserved membrane protein	2.53
Rv0220	probable esterase LipC	4.10
Rv0230c	probable phosphotriesterase php	3.98
Rv0277c	hypothetical protein	5.11
Rv0299	hypothetical protein	2.64
Rv0308	probable conseved integral membrane protein	4.24
Rv0337c	aspartate aminotransferase	4.23
Rv0349	hypothetical protein	2.74
Rv0353	heat shock protein transcriptional repressor RspR	2.49
Rv0407	f420-dependent glucose-6-phosphate dehydrogenase	2.73
Rv0436c	cdp-diacylglycerol-serine o-phosphatidyltransferase	3.54
Rv0441c	hypothetical protein	4.31
Rv0461	probable transmembrane protein	2.79
Rv0535	5'-methylthioadenosine phosphorylase	2.43
Rv0557	mannosyltransferase PimB	2.63
Rv0568	possible cytochrome p450 135b1 cyp135b1	2.55

Rv0635	hypothetical protein	4.25
Rv0642c	methoxy mycolic acid synthase 4 MmaA4	2.92
Rv0668	DNA-directed RNA polymerase beta' subunit	5.82
Rv0670	endonuclease IV	3.40
Rv0684	elongation factor ef-2	2.37
Rv0685	elongation factor tu	4.21
Rv0697	probable dehydrogenase	3.03
Rv0702	50s ribosomal protein l4	3.05
Rv0704	50s ribosomal protein l2	4.33
Rv0714	50s ribosomal protein l14	2.85
Rv0715	50s ribosomal protein l24	2.46
Rv0716	50s ribosomal protein l5	3.26
Rv0718	30s ribosomal protein s8	3.99
Rv0719	50s ribosomal protein l6	3.18
Rv0723	50s ribosomal protein l15	3.40
Rv0831c	hypothetical protein	3.89
Rv0883c	hypothetical protein	2.81
Rv0952	succinyl-coa synthetase alpha subunit	4.73
Rv0953c	possible oxidoreductase	2.84
Rv0996	probable conserved transmembrane protein	2.86
Rv1013	acyl-CoA synthase	4.34

Rv1080c	transcription elongation factor GreA	5.62
Rv1177	probable ferredoxin FdxC	2.58
Rv1201c	probable transferase	2.67
Rv1211	hypothetical protein	3.64
Rv1303	hypothetical protein	2.91
Rv1323	acetyl-CoA acetyltransferase	4.41
Rv1363c	possible membrane protein	2.57
Rv1388	putative integration host factor mihf	3.94
Rv1410c	aminoglycosides/tetracycline-transport integral membrane	2.67
Rv1422	hypothetical protein	2.48
Rv1435c	probable conserved pro, gly, val-rich secreted protein	4.31
Rv1462	hypothetical protein	4.54
Rv1545	hypothetical protein	3.95
Rv1641	translation initiation factor if-3	3.41
Rv1642	50s ribosomal protein l35	3.10
Rv1785c	probable cytochrome p450 143 cyp143	4.29
Rv1787	ppe family protein	4.76
Rv1789	ppe family protein	4.40
Rv1791	pe family protein	2.68
Rv1793	putative esat-6 like protein esxn (esat-6 like protein 5)	3.10
Rv1794	hypothetical protein	3.25

Rv1796	probable proline rich membrane-anchored mycosin mycp5	2.45
Rv1830	hypothetical protein	4.38
Rv1846c	possible transcriptional regulatory protein	2.42
Rv1916	isocitrate lyase	4.06
Rv1926c	immunogenic protein mpt63	2.77
Rv1932	thiol peroxidase	5.28
Rv2054	hypothetical protein	3.68
Rv2094c	twin argininte translocase protein a	2.85
Rv2177c	possible transposase	3.02
Rv2196	probable ubiquinol-cytochrome c reductase qcrb	2.91
Rv2199c	possible conserved integral membrane protein	3.49
Rv2200c	probable transmembrane cytochrome c oxidase ctac	3.82
Rv2220	glutamine synthetase glna1 (glutamine synthase)	4.76
Rv2258c	possible transcriptional regulatory protein	2.44
Rv2376c	low molecular weight antigen cfp2	3.61
Rv2428	alkyl hydroperoxide reductase c protein ahpc	3.89
Rv2468c	hypothetical protein	3.68
Rv2469c	hypothetical protein	2.53
Rv2584c	adenine phosphoribosyltransferase	2.67
Rv2586c	protein export protein secf	3.22
Rv2617c	probable transmembrane protein	2.87

Rv2621c	possible transcriptional regulatory protein	2.42
Rv2676c	hypothetical protein	3.90
Rv2723	probable conserved integral membrane protein	2.65
Rv2783c	polynucleotide phosphorylase, pnpase	4.13
Rv2861c	methionine aminopeptidase	2.89
Rv2888c	amidase	2.39
Rv2889c	elongation factor ts	3.97
Rv2949c	hypothetical protein	3.73
Rv2950c	acyl-coa synthase	4.88
Rv2954c	hypothetical protein	3.21
Rv3139	probable acyl-coa dehydrogenase fade24	3.94
Rv3157	nadh dehydrogenase subunit m	4.22
Rv3211	probable atp-dependent rna helicase rhle	4.22
Rv3222c	hypothetical protein	3.09
Rv3246c	two component regulatory protein mtra	2.90
Rv3248c	s-adenosyl-l-homocysteine hydrolase	3.74
Rv3277	probable conserved transmembrane protein	2.43
Rv3280	probable propionyl-coa carboxylase beta chain 5 accd5	3.48
Rv3281	hypothetical protein	2.96
Rv3299c	probable arylsulfatase atsb	3.07
Rv3456c	50s ribosomal protein l17	5.00

Rv3460c	30s ribosomal protein s13	2.91
Rv3461c	50s ribosomal protein l36	2.76
Rv3465	"dtdp-4-dehydrorhamnose 3,5-epimerase rmlc	3.07
Rv3496c	mce-family protein Mce4D	2.37
Rv3513c	probable fatty-acid-coa ligase fadd18	2.39
Rv3587c	probable conserved membrane protein	2.67
Rv3596c	atp-dependent protease atp-binding subunit clpc1	3.61
Rv3597c	probable iron-regulated lsr2 protein precursor	2.88
Rv3611	hypothetical arginine and proline rich protein	2.42
Rv3613c	hypothetical protein	4.41
Rv3614c	hypothetical protein	6.03
Rv3623	probable conserved lipoprotein lpqg	2.57
Rv3723	probable conserved transmembrane protein	2.54
Rv3780	hypothetical protein	4.43
Rv3798	probable transposase	3.29
Rv3804c	secreted antigen 85-a fbpa (antigen 85 complex a)	4.69
Rv3846	superoxide dismutase [fe] sodA	4.00
Rv3850	hypothetical protein	2.39
Rv3914	thioredoxin trxc (trx) (mpt46)	2.48

[a] Annotated functions provided by TubercuList and BCGList.

Table S3. Genes significantly under-represented 2-fold or more in intracellular *fdr8* compared to intracellular mc[2]6455 (wild type), 3 days post THP-1 cell infection.

ORF	Gene Function [a]	Fold change compared to wild type
MT1775	hypothetical protein	−3.40
MT1931.1	hypothetical protein	−6.01
MT2045	hypothetical protein	−4.54
MT2094	transcriptional regulator, ArsR family	−4.45
MT2174	hypothetical protein	−5.41
Rv0058	replicative DNA helicase	−3.07
Rv0065	hypothetical protein	−2.99
Rv0108c	hypothetical protein	−3.40
Rv0118c	hypothetical protein	−3.84
Rv0232	probable TetR/AcrR transcriptional regulator	−3.48
Rv0251c	heat shock protein hsp	−2.88
Rv0264c	hypothetical protein	−5.30
Rv0476	possible conserved transmembrane protein	−2.98
Rv0539	probable dolichyl-phosphate sugar synthase	−3.28
Rv0560c	possible benzoquinone methyltransferase	−3.52
Rv0744c	possible transcriptional regulatory protein	−2.92
Rv0789c	hypothetical protein	−3.02
Rv0847	probable lipoprotein LpqS	−4.44

Rv0871	probable cold shock-like protein B CspB	−2.96
Rv0990c	hypothetical protein	−5.99
Rv1029	potassium-transporting ATPase subunit A	−4.02
Rv1059	hypothetical protein	−4.36
Rv1232c	hypothetical protein	−4.30
Rv1406	methionyl-tRNA formyltransferase	−3.17
Rv1443c	hypothetical protein	−3.70
Rv1510	conserved probable membrane protein	−3.02
Rv1535	hypothetical protein	−2.92
Rv1588c	Partial REP13E12 repeat protein	−3.05
Rv1678	probable integral membrane protein	−3.49
Rv1907c	hypothetical protein	−2.90
Rv1909c	ferric uptake regulation protein FurA	−5.52
Rv1952	hypothetical protein	−3.58
Rv1989c	hypothetical protein	−3.90
Rv1990c	probable transcriptional regulatory protein	−4.13
Rv2020c	hypothetical protein	−3.93
Rv2021c	possible transcriptional regulatory protein	−3.19
Rv2022c	hypothetical protein	−3.31
Rv2034	Probable ArsR-type repressor protein	−5.49
Rv2036	hypothetical protein	−3.72

Rv2558	hypothetical protein	−4.23
Rv2694c	hypothetical protein	−3.08
Rv2745c	possible transcriptional regulatory protein	−3.37
Rv2926c	hypothetical protein	−2.94
Rv3250c	probable rubredoxin RubB	−3.28
Rv3252c	probable transmembrane alkane 1-monooxygenase AlkB	−3.54
Rv3270	probable metal cation-transporting p-type atpase c ctpc	−2.43
Rv3295	probable transcriptional regulatory protein, tetr-family	−2.96
Rv3473c	possible peroxidase BpoA	−3.51
Rv3546	acetyl-CoA acetyltransferase	−2.95
Rv3557c	probable transcriptional regulatory protein TetR-family	−3.60
Rv3705c	hypothetical protein	−4.69
Rv3714c	hypothetical protein	−5.14
Rv3753c	hypothetical protein	−4.71
Rv3841	possible bacterioferritin bfrB	−2.27

[a] Annotated functions provided by TubercuList and BCGList.

Table S4. Genes significantly over-represented in *fdr8*-infected THP-1 cells, compared to mc²6455 (BCG parent) -infected THP-1 cells, 3 days post infection.

Gene	mRNA Accession [a]	mRNA Description [b]	Fold-change
MIR223	NR_029637	microRNA 223 (MIR223), microRNA.	2.01
MIRLET7F1	NR_029483	microRNA let-7f-1 (MIRLET7F1), microRNA.	1.92
THBS1	NM_003246	thrombospondin 1 (THBS1).	1.63
UQCRB	NM_006294	ubiquinol-cytochrome c reductase binding protein (UQCRB), nuclear gene encoding mitochondrial protein	1.60
VCAM1	NM_001078	vascular cell adhesion molecule 1 (VCAM1), transcript variant 1	1.59
MIR24-2	NR_029497	microRNA 24-2 (MIR24-2), microRNA.	1.56
TRIM52	NM_032765	tripartite motif-containing 52 (TRIM52)	1.53
WEE1	BX641032	cDNA DKFZp686I18166	1.52
C5orf54	NM_022090	chromosome 5 open reading frame 54	1.50
MIR17HG	NR_027350	MIR17 host gene (non-protein coding) (MIR17HG), non-coding RNA.	1.49
MIR222	NR_029636	microRNA 222 (MIR222), microRNA.	1.49
CXCR5	NM_001716	chemokine (C-X-C motif) receptor 5 (CXCR5), transcript variant 1	1.48
RRP7B	BC014647	cDNA clone IMAGE:4646900.	1.48
LOC100131860	AK097109	cDNA FLJ39790 fis, clone SPLEN2003204.	1.46
CXCL6	NM_002993	chemokine (C-X-C motif) ligand 6 (granulocyte chemotactic protein 2) (CXCL6)	1.42
MIR15A	NR_029485	microRNA 15a (MIR15A), microRNA.	1.41

CCL20	NM_004591	chemokine (C-C motif) ligand 20 (CCL20), transcript variant 1	1.40
MIR146A	NR_029701	microRNA 146a (MIR146A), microRNA.	1.40
AREG	NM_001657	amphiregulin (AREG)	1.39
C3orf78	NM_001124767	chromosome 3 open reading frame 78 .	1.38
SNHG12	AY277594	transformation-related protein 11 (TRG11)	1.38
GFPT2	NM_005110	glutamine-fructose-6-phosphate transaminase 2 (GFPT2)	1.37
SHROOM3	NM_020859	shroom family member 3 (SHROOM3)	1.35
LOC100130428	BC040288	Homo sapiens cDNA clone IMAGE:4822341.	1.35
PELO	NM_015946	pelota homolog (Drosophila) (PELO)	1.35
FAM36A	NM_198076	family with sequence similarity 36, member A	1.34
MIRLET7G	NR_029660	microRNA let-7g (MIRLET7G), microRNA.	1.33
KCNRG	NM_173605	potassium channel regulator, transcript variant 1	1.32
TNFAIP6	NM_007115	tumor necrosis factor, alpha-induced protein 6	1.32
DUSP5P	AK055963	cDNA FLJ31401 fis, clone NT2NE2000038.	1.32
GRIK2	NM_175768	glutamate receptor, ionotropic, kainate 2 transcript variant 2	1.31
DUSP5P	NR_002834	dual specificity phosphatase 5 pseudogene, non-coding RNA.	1.31
EHD1	NM_006795	EH-domain containing 1 (EHD1)	1.30
CXCL1	NM_001511	chemokine (C-X-C motif) ligand 1 (melanoma growth stimulating activity, alpha)	1.30

SLC7A2	NM_003046	solute carrier family 7 (cationic amino acid transporter, y+ system), member 2, transcript var 1	1.29
MIR30E	NR_029846	microRNA 30e (MIR30E), microRNA.	1.28
PSTPIP2	NM_024430	pro-ser-thre phosphatase interacting protein 2	1.28
GPR141	NM_181791	G protein-coupled receptor 141 (GPR141)	1.28
TTC14	NM_133462	tetratricopeptide repeat domain 14, transcript var 1	1.28
PARM1	NM_015393	prostate androgen-regulated mucin-like protein 1	1.27
DCAF16	NM_017741	DDB1 and CUL4 associated factor 16 (DCAF16)	1.27
AIDA	NM_022831	axin interactor, dorsalization associated (AIDA)	1.26
RBM14	NM_006328	RNA binding motif protein 14 (RBM14)	1.26
CCL4L1	NM_001001435	chemokine (C-C motif) ligand 4-like 1	1.26
CCL4	NM_002984	chemokine (C-C motif) ligand 4,transcript variant 1	1.26
CCL19	NM_006274	chemokine (C-C motif) ligand 19 (CCL19)	1.25
SVIP	NM_148893	small VCP/p97-interacting protein (SVIP)	1.25
NCF1	NM_000265	neutrophil cytosolic factor 1 (NCF1)	1.24
SLC7A11	NM_014331	solute carrier family 7, (cationic amino acid transporter, y+ system) member 11	1.24
NCF1	NM_000265	neutrophil cytosolic factor 1 (NCF1)	1.24
NCF1	NM_000265	neutrophil cytosolic factor 1 (NCF1)	1.23
CCL4L1	NM_001001435	chemokine (C-C motif) ligand 4-like 1 (CCL4L1)	1.22
CCL4L1	NM_001001435	chemokine (C-C motif) ligand 4-like 1 (CCL4L1)	1.22
WRN	NM_000553	Werner syndrome, RecQ helicase-like (WRN)	1.22

NBPF4	NM_001143989	neuroblastoma breakpoint family, member 4	1.22
ARGLU1	NM_018011	arginine and glutamate rich 1 (ARGLU1)	1.22
NBPF6	NM_001143987	neuroblastoma breakpoint family, member 6, transcript variant 1	1.21
MAP1LC3A	NM_032514	microtubule-associated protein 1 light chain 3 alpha, transcript variant 1	1.21
COL8A1	NM_001850	collagen, type VIII, alpha 1, transcript variant 1	1.20
OGT	NM_181672	O-linked N-acetylglucosamine (GlcNAc) transferase (UDP-N-acetylglucosamine: polypeptide-N-acetylglucosaminyl transferase), transcript variant 1	1.19
CRIM1	NM_016441	cysteine rich transmembrane BMP regulator 1 (chordin-like)	1.18
IL1B	NM_000576	interleukin 1, beta (IL1B)	1.18
ZCCHC5	NM_152694	zinc finger, CCHC domain containing 5 (ZCCHC5)	1.18
PDGFRA	NM_006206	platelet-derived growth factor receptor, alpha polypeptide	1.17
GLMN	NM_053274	glomulin, FKBP associated protein (GLMN)	1.17
CYP7B1	NM_004820	cytochrome P450, family 7, subfamily B, polypep1	1.16
MFSD8	NM_152778	major facilitator superfamily domain containing 8	1.16

[a] mRNA accession numbers for human genes provided by UCSC Browser [112].

[b] Predicted functions provided by IPA and UCSC Browser.

Table S5. Genes significantly under-represented in *fdr8*-infected THP-1 cells, compared to mc[2]6455 (BCG parent) -infected THP-1 cells, 3 days post infection.

Gene	mRNA Accession [a]	mRNA Description [b]	Fold-change
CHIT1	NM_003465	chitinase 1 (chitotriosidase) (CHIT1)	−1.64
METTL7B	NM_152637	methyltransferase like 7B (METTL7B)	−1.61
ITGA3	NM_002204	integrin, alpha 3 (antigen CD49C, alpha 3 subunit of VLA-3 receptor) (ITGA3), transcript variant a	−1.54
MMP7	NM_002423	matrix metallopeptidase 7 (MMP7)	−1.53
RNASE1	NM_198232	ribonuclease, RNase A family, 1 (pancreatic) (RNASE1), transcript variant 3	−1.51
MSR1	NM_002445	macrophage scavenger receptor 1 (MSR1), transcript variant SR-AII	−1.46
DYSFIP1	NM_001007533	dysferlin interacting protein 1 (DYSFIP1)	−1.44
CD180	NM_005582	CD180 molecule (CD180)	−1.43
NYNRIN	NM_025081	NYN domain and retroviral integrase containing (NYNRIN)	−1.42
VWA5A	NM_001130142	von Willebrand factor A domain containing 5A (VWA5A), transcript variant 3	−1.40
FADS2	NM_004265	fatty acid desaturase 2 (FADS2)	−1.33
GAS6	NM_000820	growth arrest-specific 6 (GAS6), transcript variant 1	−1.25

[a] mRNA accession numbers for human genes provided by UCSC Browser [112].

[b] Predicted functions provided by IPA and UCSC Browser.

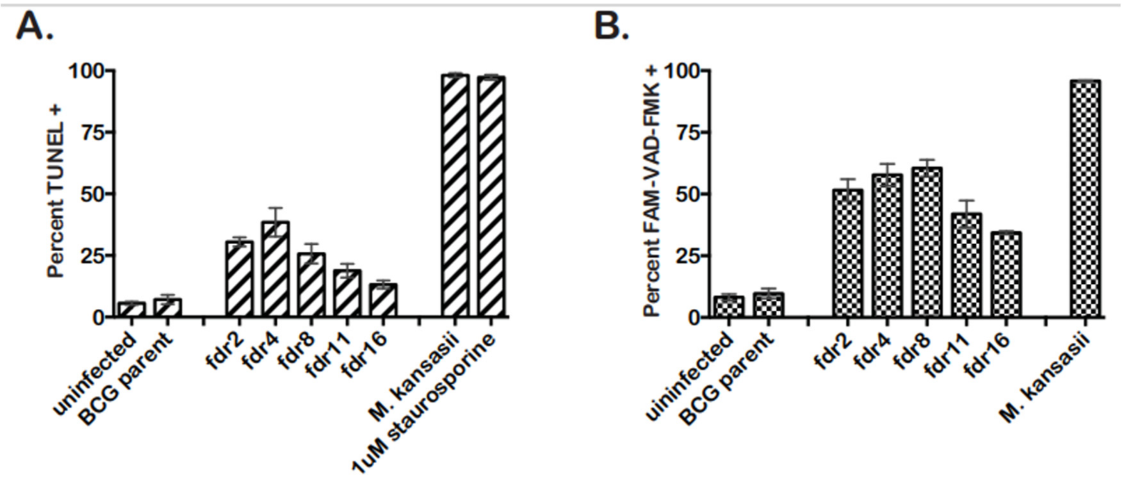

Figure S1. Preliminary cell death analyses of *fdr*-infected THP-1 cells. A. *fdr*-infected THP-1 cells are TUNEL positive. THP-1 cells were infected at an MOI 5:1 with mycobacteria strains. On day 3, cells were analyzed by TUNEL staining and fluorescence microscopy. B. *fdr*-infected THP-1 cells show activated caspases. Similarly infected THP-1 cells were stained on day 3 for poly-caspase activity and analyzed by microscopy. For both A. and B., cells were counted at 200X from three different fields, 100 cells per field. Data are shown from two independent experiments. For *fdr* infections * $P < 0.05$ relative to BCG parent control.

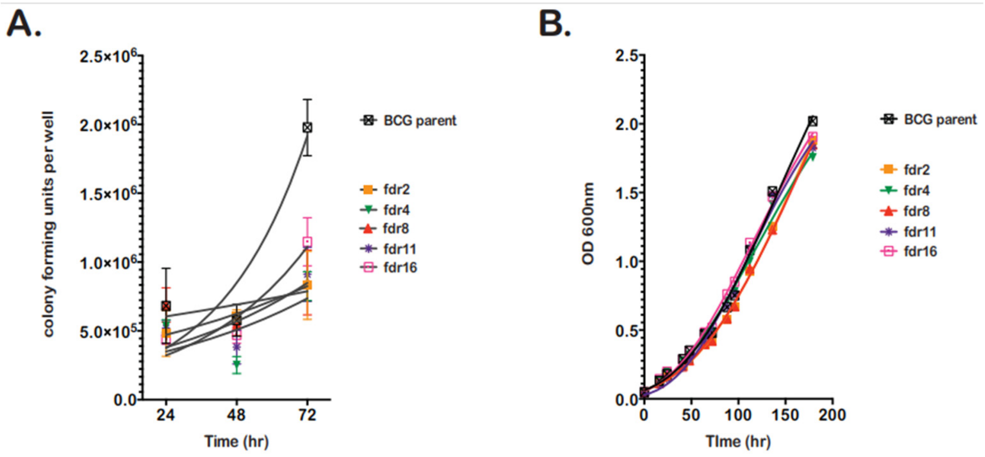

Figure S2. *In vitro* and intracellular growth characteristics of *fdr* mutants. A. *fdr* mutants show reduced cfu in THP-1 MΦ 3 days post-infection. THP-1 cells were infected with mycobacteria at an MOI 5:1 and 3 days post-infection, lysed in 0.05% SDS. Lysates were serial diluted in H_2O from 100-10-7 and spotted for cfus. 14 days later, mycobacteria colonies were counted. Data are averaged from three independent experiments. For *fdr* population *** $P < 0.001$, relative to BCG parent control. B. *fdr* mutants do not have a growth defect in complete 7H9 media. Mid-log phase cultures were inoculated to an OD600nm = 0.05 in complete 7H9 broth and monitored over 7 days for growth. Results shown are representative of two independent experiments with similar results. For all *fdr* mutants: not significant, relative to BCG parent control.

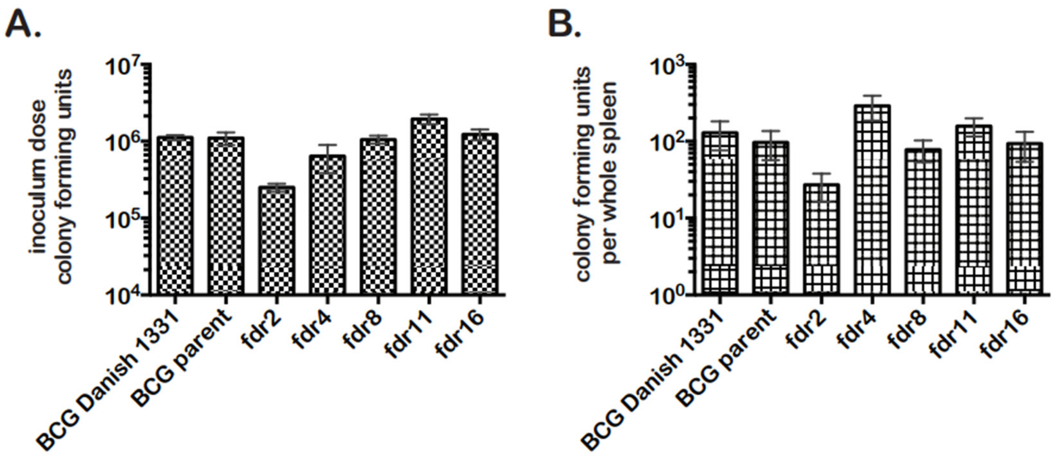

Figure S3. ***fdr*-mutants enhance *M. tuberculosis*-specific CD4 and CD8 T cell responses *in vivo*.** C57BL/6 mice were subcutaneously immunized with *fdr* mutants or controls, and 3 weeks post-immunization, spleens harvested for IFN-γ ELISPOT. A. Inoculum dose of bacterial strains used for ELISPOT experiment. B. Bacterial burden in spleen at harvest. Data are from one experiment, 3 mice per group; means and standard deviations are shown.

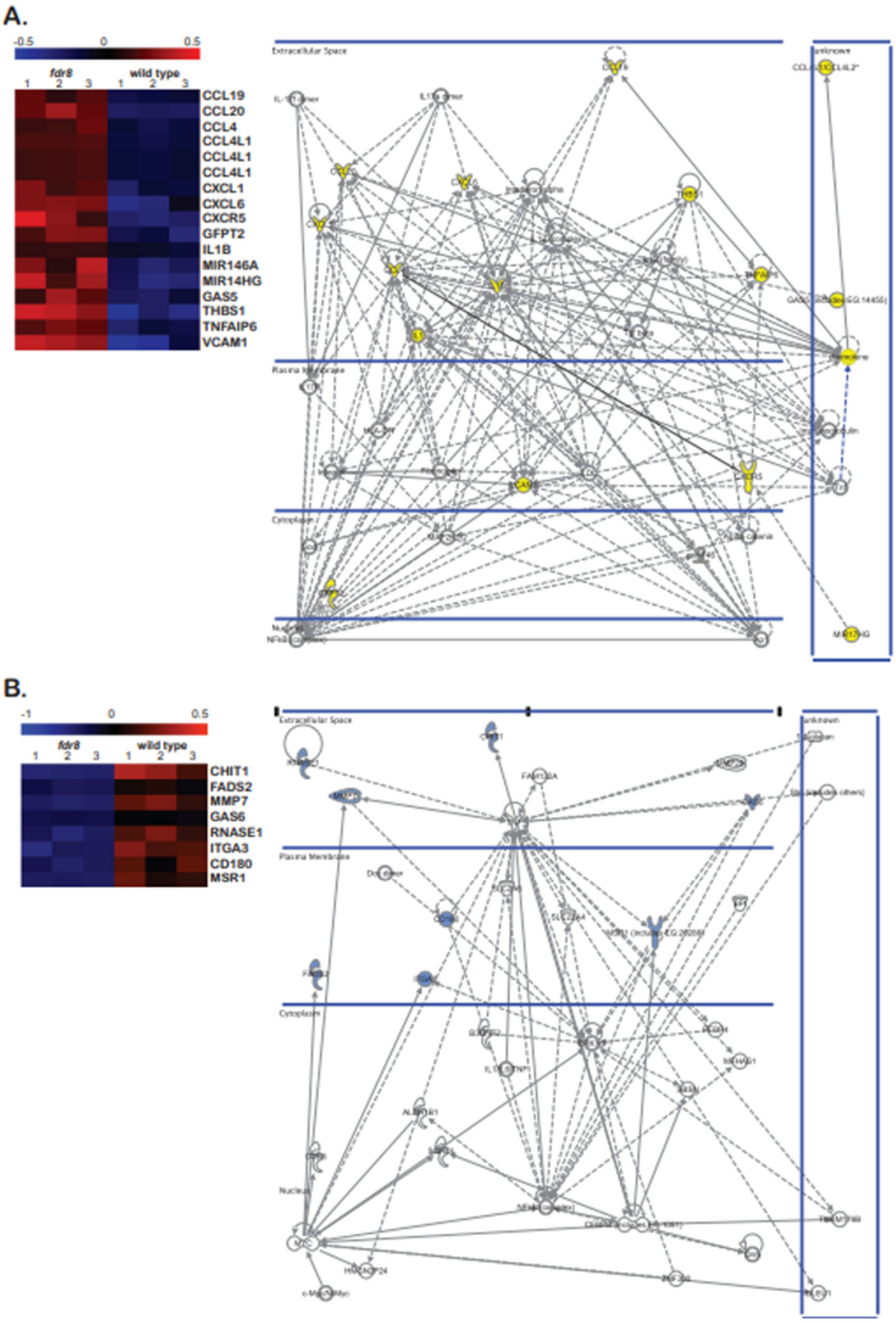

Figure S4. MΦ functional pathways modulated by *fdr8*. A. Significantly over-represented host genes in *fdr8*-infected THP-1 cells and Ingenuity Systems pathway analysis of significantly over-represented genes in *fdr8*-infected THP-1 cells, compared to BCG parent-infected control. Left: Mean-weighted expression of genes significantly over-represented in *fdr8*-infected THP-1 cells and part of the top enriched IPA biological network. Right: Top biological network significantly over-represented in *fdr8*-infected THP-1 cells. Genes identified by microarray are indicated in yellow. IPA provided subcellular localizations and interactions shown: solid lines indicate known protein-protein interactions, dotted lines indicate known expression relationships. B. Significantly under-represented host genes in *fdr8*-infected THP-1 cells and Ingenuity Systems pathway analysis of significantly under-represented genes in *fdr8*-infected THP-1 cells, compared to BCG parent-infected control. Left: Mean-weighted expression of genes significantly under-represented in *fdr8*-infected THP-1 cells and part of the top enriched IPA biological network. Right: Top biological network significantly under-represented in *fdr8*-infected THP-1 cells. Genes identified by microarray are indicated in blue. IPA-provided subcellular localizations and interactions shown: solid lines indicate known

protein-protein interactions, dotted lines indicate known expression relationships. All microarray results were analyzed using TIGR MeV software.

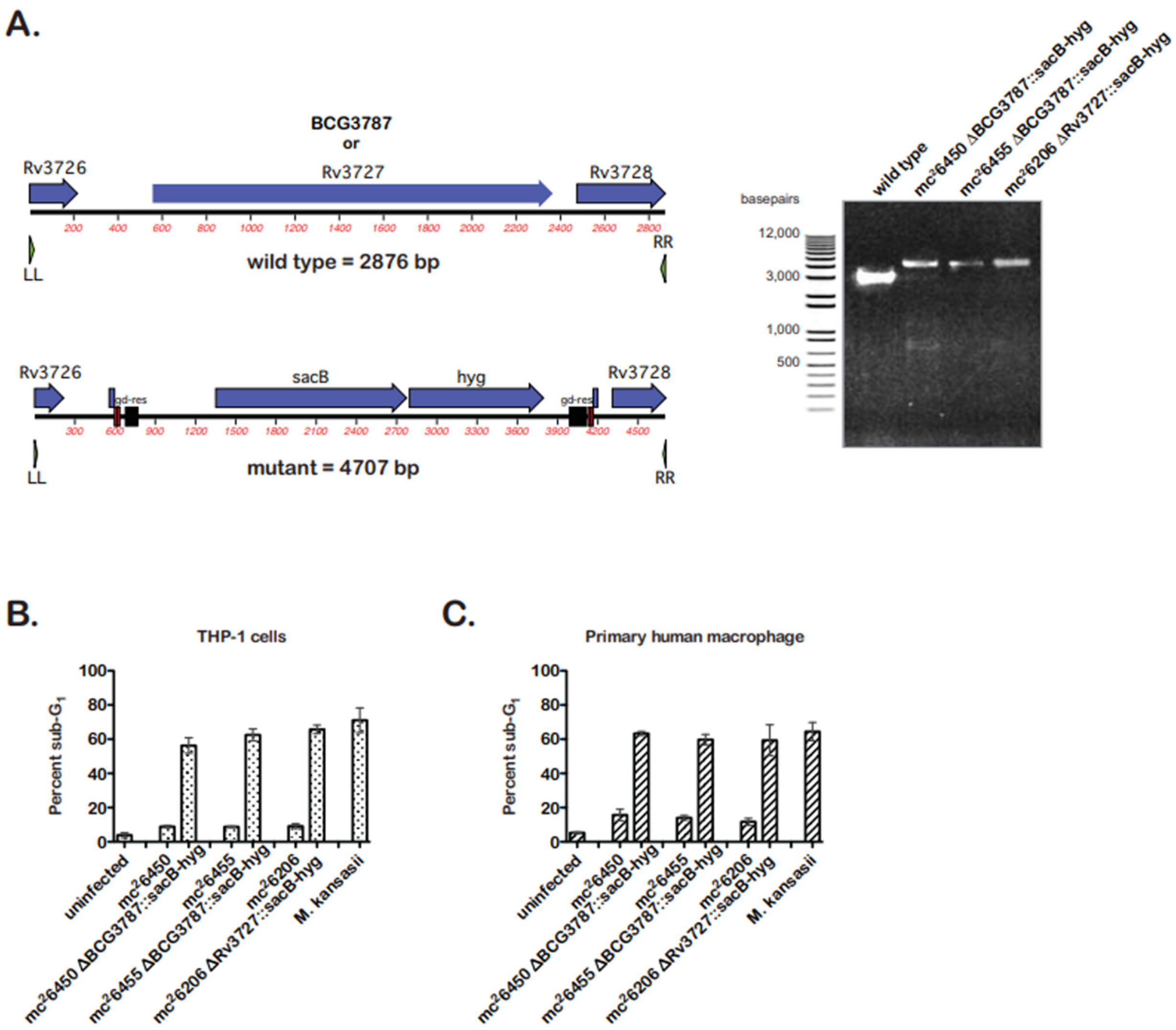

Figure S5. Precise gene deletion of *fdr8* in *M. bovis* BCG Danish and *M. tuberculosis ΔpanCD ΔleuCD* induce pyroptosis of MΦ. A. Expected PCR amplicon sizes are indicated for wild type and mutant strains (left panel). Confirmation of gene knockout by PCR is shown (right panel); wild type is mc²6450 (BCG Danish strain 1331) genomic DNA. B. Rv3727 null -induced pyroptosis in THP-1 cells. Mycobacteria strains were used to infect THP-1 cells at an MOI = 5:1, and 3 days later cells analyzed for cell death by sub-G_1 staining and flow cytometry. C. Rv3727 null -induced pyroptosis in primary human MΦ. Similarly infected primary human MΦ were analyzed for cell death by sub-G_1 staining and flow cytometry. For both B. and C., data are from three independent experiments. For all *fdr* infections *** $P < 0.001$, relative to wild type control of each genetic background.

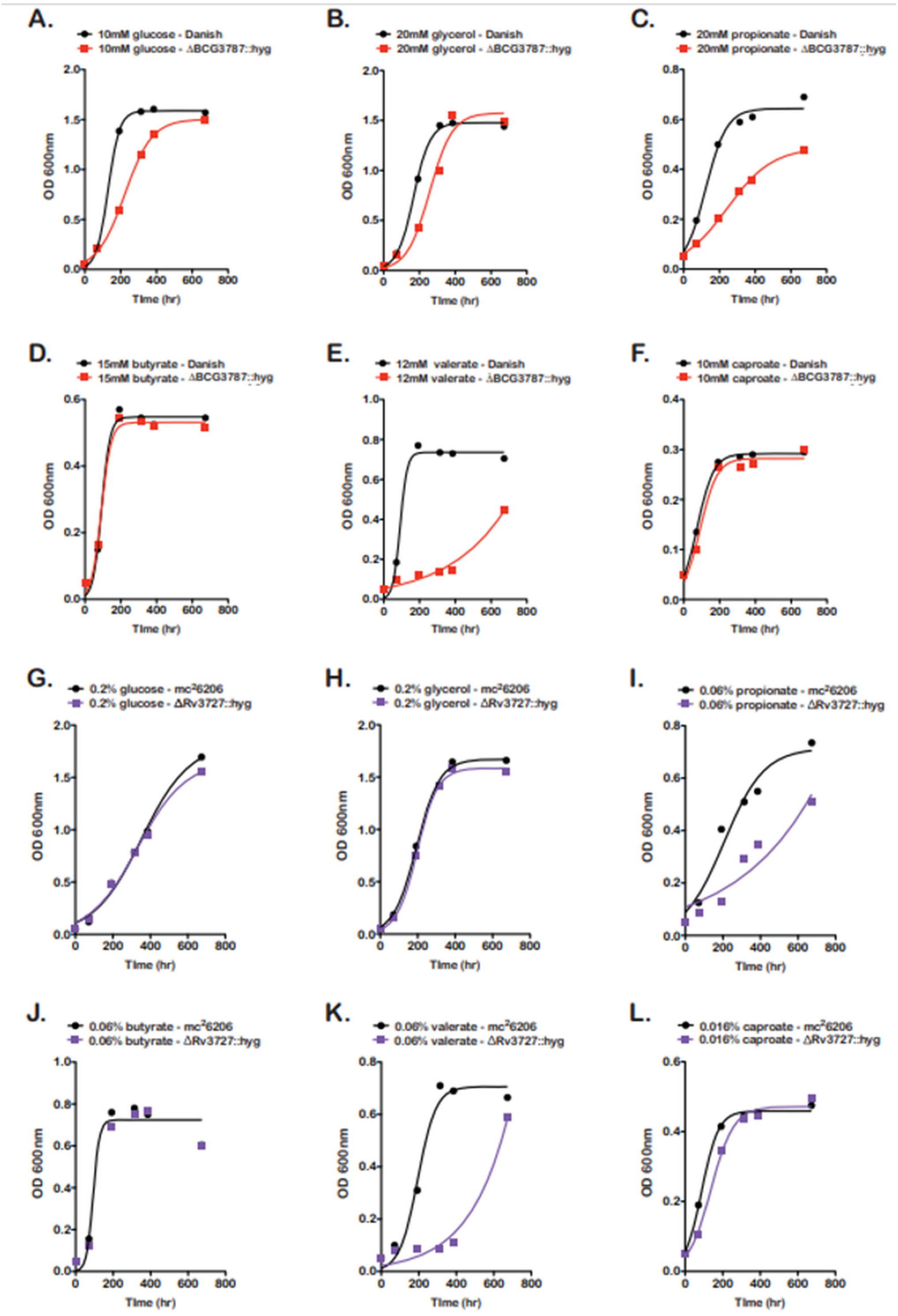

Figure S6. ***fdr8*** **null mutants are defective for growth in odd-chain fatty acids.** Mid-log phase bacteria were washed and resuspended to an OD_{600nm} of 0.05 in media containing different carbon sources. Growth was monitored by OD_{600nm} over 4 weeks. Growth curves are shown for *M. bovis* BCG Danish strains grown in A. glucose, B. glycerol, C. propionate, D. butyrate, E. valerate, F. caproate. Growth curves are shown for *M. tuberculosis ΔpanCD ΔleuCD* strains grown in G. glucose, H. glycerol, I. propionate, J. butyrate, K. valerate, L. caproate. Logistic regression curve fitting analysis was done using GraphPad Prism 5 and used to calculate grown rate (k, see TABLE 3). Results are representative of two independent experiments with similar results. .
